# Imaging oxygen metabolism with hyperpolarized magnetic resonance: a novel approach for the examination of cardiac and renal function

**DOI:** 10.1042/BSR20160186

**Published:** 2017-01-27

**Authors:** Marie Schroeder, Christoffer Laustsen

**Affiliations:** MR Research Centre, Department of Clinical Medicine, Aarhus University, Aarhus, Denmark

**Keywords:** hyperpolarization, MRI, metabolism, oxygen

## Abstract

Every tissue in the body critically depends on meeting its energetic demands with sufficient oxygen supply. Oxygen supply/demand imbalances underlie the diseases that inflict the greatest socio-economic burden globally. The purpose of this review is to examine how hyperpolarized contrast media, used in combination with MR data acquisition methods, may advance our ability to assess oxygen metabolism non-invasively and thus improve management of clinical disease. We first introduce the concept of hyperpolarization and how hyperpolarized contrast media have been practically implemented to achieve translational and clinical research. We will then analyse how incorporating hyperpolarized contrast media could enable realization of unmet technical needs in clinical practice. We will focus on imaging cardiac and renal oxygen metabolism, as both organs have unique physiological demands to satisfy their requirements for tissue oxygenation, their dysfunction plays a fundamental role in society’s most prevalent diseases, and each organ presents unique imaging challenges. It is our aim that this review attracts a multi-disciplinary audience and sparks collaborations that utilize an exciting, emergent technology to advance our ability to treat patients adversely affected by an oxygen supply/demand mismatch.

## Introduction

Oxygen metabolism – the delivery of oxygen to tissues for the purpose of facilitating efficient breakdown of energetic substrates into ATP – is fundamental for the healthy function of all tissues in the body. For efficient oxygen metabolism to take place, a number of steps must occur continuously [1]:

1. Oxygen must be delivered in sufficient amounts to a given tissue via blood flow and perfusion (oxygen supply).
2. Oxygen must be extracted efficiently from haemoglobin in the blood to the cells where it is required (oxygen availability).
3. Oxygen must be utilized in combination with the biochemical machinery in place and energetic substrate to produce ATP (oxygen consumption).

The outcome of successful oxygen metabolism in an organ is ample energy to power all of that organ’s specific physiological functions.

As the anatomy and energetic requirements of each organ in the body vary widely, the physiological processes driving oxygen metabolism also vary among organs. What is true throughout the body, however, is that a defect in oxygen metabolism that prevents an organ from generating sufficient ATP probably is a cause or consequence of clinical disease. Imbalances in oxygen supply and demand underlie the two clusters of diseases that inflict the greatest socio-economic burden globally: cardiovascular diseases (particularly ischaemic heart disease) and diabetes [2]. For example, impaired oxygen supply to the heart or kidney is the initiating event of the ischaemic cascade, which if not treated appropriately, can lead to compromised tissue function and potentially life-threatening cell death. Chronic ischaemia resulting from microvascular dysfunction has been implicated as both a cause and consequence of diabetes, heart failure with preserved ejection fraction (HFpEF) and chronic kidney disease [3–5]. Moreover, reduced oxygen consumption in the context of ample supply can be indicative of impaired tissue function in diseases including hibernating myocardium [6] and heart failure [1].

The centrality of oxygen metabolism to maintain human health implies that the ability to measure it accurately as a routine aspect of clinical practice is essential. Robust and accurate measurements of blood flow, perfusion, tissue oxygenation and oxygen consumption may lead to new diagnostic strategies and could facilitate the evaluation of therapies designed to improve oxygen supply/demand imbalances or to support energy metabolism [1]. Significant multi-modality advances have been made to non-invasive methods capable of imaging absolute oxygen metabolism. To date, positron emission tomography (PET) has been used as a gold standard to provide quantitative measurements of oxygen supply and oxygen consumption, with ^15^O-water considered an ideal diffusible agent to measure perfusion [7] and ^11^C-acetate to determine metabolic oxygen consumption [1,8]. Ionizing radiation, long scan times, limited availability, requirement of an onsite cyclotron and high costs have restricted use of these PET techniques to single time-point assessments performed at experienced research centres. In contrast, magnetic resonance (MR) offers many benefits in safety and cost that make its routine use in the clinic viable [9]. Moreover, the versatility of MR hardware and software implies that multiple anatomical and functional parameters could be measured in a single scanning session.

MRI encounters difficulties in imaging heterogeneous tissues that experience respiratory and cardiac motion, and in characterizing oxygen consumption. MR perfusion imaging, for example, tends to rely on gadolinium-based contrast agents that are contraindicated in patients with renal insufficiency [10], and MRS methods to assess substrate metabolism and ATP production require specialized implementation and long scan times. It is the advent of hyperpolarization, a technique to generate high-MR signal media, which is poised to advance MR techniques to a veritable ‘one-stop shop’ for imaging oxygen metabolism. Hyperpolarization using the dynamic nuclear polarization technique (DNP) can yield >10000-fold signal increases in MR-active nuclei [11]. The spatial distribution of hyperpolarized media can be imaged for the assessment of oxygen supply, whereas the breakdown of energetic substrates can be monitored using MR to allow unprecedented visualization of the oxidative formation of ATP [12,13]. The potential of these tracers has been demonstrated extensively in pre-clinical studies, and the first patient studies are underway at leading research centres around the world [14,15].

The purpose of this review is to critically examine how hyperpolarized media, used in combination with MR data acquisition methods, may advance our ability to assess oxygen metabolism non-invasively and thus improve management of clinical disease. To achieve this, we will first introduce the concept of hyperpolarization and how hyperpolarized contrast media have been practically implemented to achieve translational and clinical research. We will then introduce how MR studies of oxygen metabolism are commonly performed, and analyse how incorporating hyperpolarized contrast media could enable realization of unmet technical needs in clinical practice in the near future. We will focus on imaging cardiac and renal oxygen metabolism, as both organs have unique physiological demands to satisfy their requirements for tissue oxygenation, their dysfunction plays a fundamental role in society’s most prevalent diseases and each organ presents unique imaging challenges. It is our aim that this review attracts a multi-disciplinary audience and sparks collaborations that utilize an exciting, emergent technology to advance our ability to treat patients adversely affected by an oxygen supply/demand mismatch.

## Production of hyperpolarized contrast media

Hyperpolarization overcomes the low sensitivity inherent to MRI to generate huge contrast to noise for imaging and spectroscopy applications. DNP, when applied to an exogenous dose of a compound containing MR-active nuclei such as ^13^C or ^1^H, temporarily aligns all nuclei in the same direction to increase the polarization and hence the signal of a particular compound. The DNP technique as it pertains to physiological study has been reviewed extensively elsewhere (e.g. [12,16]), so here we introduce it briefly. It relies on the fact that electrons have a very high level of polarization (approaching 100%) at temperatures near absolute zero and in a magnetic field. To employ the DNP process, a free radical is mixed with an MR-active tracer of interest in an environment of a strong magnetic field (3–5 T) and supercooled liquid helium (1–2 K), and the electronic polarization to the tracer of interest through the use of microwave irradiation. Depending on the molecule being polarized, this process typically takes between 30 and 120 min and results in a nuclear polarization of up to 50%. Such polarization levels are much greater than the normal MRI polarization levels on the order of 0.0005%.

It is necessary to bring the sample up from 1 K to a physiological temperature before use in an *in vivo* experiment. To achieve this, the hyperpolarized sample is rapidly dissolved by the injection of a heated and pressurized bolus of aqueous solvent. The resulting solution retains a high level of nuclear polarization (approximately 20–40%) and can be formulated to be at physiological temperature, osmolarity and pH for *in vivo* injection. The polarization produced then steadily decays back to the normal thermal equilibrium level at a rate dependent on the inherent relaxation properties of the molecule under study (typically 1–2 min). Thus, a current limitation of the method is that the enhanced signal is available for only a short period of time. Unique, fast MR acquisition strategies have been developed to make the best use of the hyperpolarized signal available in a range of applications [17–23].

Theoretically, the DNP technique can be applied to a wide range of molecules labelled with ^13^C or any other MR-active nucleus, including ^1^H. However, for imaging of oxygen metabolism, several criteria must be met for the successful polarization and *in vivo* detection of the hyperpolarized molecule and its products. This includes limitations on the molecular properties, safety and the speed of uptake and use [24]. Overall, hyperpolarized ^13^C MR studies have used the molecules [1-^13^C]pyruvate, [2-^13^C]pyruvate, ^13^C-urea and [1,4-^13^C_2_]fumarate most frequently because they polarize efficiently, retain hyperpolarization for a relatively long time (time constants of signal decay are approximately 45 s) and probe unique aspects of cellular uptake and metabolism that are disturbed in disease.

**Figure 1:**
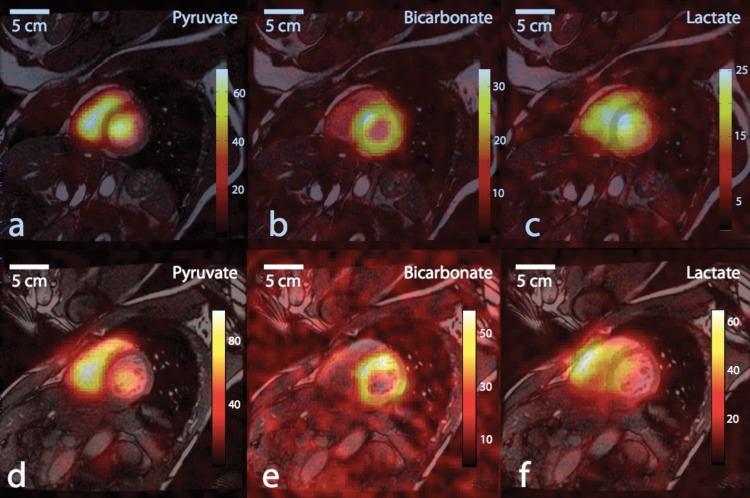
Representative cardiac ^13^C images, obtained from a healthy person, displayed as colour overlays on top of greyscale anatomical images in a mid-left ventricle (LV) slice from two subjects. The [1-^13^C]pyruvate substrate was seen mainly in the blood pool within the cardiac chambers (a,d). Flux of pyruvate through the pyruvate dehydrogenase complex (PDC) is reflected in the ^13^C-bicarbonate images (b,e), with signal predominantly in the wall of the LV. The [1-^13^C]lactate signal (c,f) appeared with a diffuse distribution covering the muscle and chambers. Reproduced from [14] with permissions from the authors and publisher.

In practice, DNP is achieved by using a stand-alone piece of equipment – a hyperpolarizer – that is situated in close proximity to the MR scanner. The hyperpolarizer has evolved through several versions of increasingly sophisticated technology over the last decade and the start-of-the-art now is the SpinLab, a system in which contrast media appropriate for injection into patients can be made. Approximately 20 sites globally have access to SpinLabs for translational research, with five sites (to our knowledge) using their SpinLabs to generate sterile hyperpolarized [1-^13^C]pyruvate for infusion into patients (cardiac images are shown in Figure 1 [25]). The majority of hyperpolarized MR research centres are focused on interrogating the [1-^13^C]pyruvate to [1-^13^C]lactate conversion to diagnose tumour severity [15,26,27] based on the so-called Warburg effect, in which in general tumour tissue is known to favour glycolytic metabolism at the expense of oxidative metabolism, regardless of the presence of oxygen [28]. Oncology studies using hyperpolarized methods have been discussed in great detail elsewhere [26], and as they mostly target non-oxidative metabolism, are outside of the scope of the present article. We will focus on studies that have developed technology or demonstrated proof-of-concept for imaging oxygen supply (angiography and perfusion), oxygen availability and oxidative substrate breakdown using hyperpolarized contrast media.

## Angiography and perfusion imaging

The imaging-based methods available for assessing blood and nutrient supply reveal several areas of unmet clinical need, as measurement of local haemodynamic changes is essential for understanding the oxygen supply and subsequent usage. Performing angiography without ionizing radiation remains challenging, and MR angiography (MRA) has been hindered by the high spatiotemporal resolution required to image stenosed arteries accurately [12,29]. Perfusion imaging without potentially toxic contrast and with quantification remains a challenge. Furthermore, robust imaging of suspected kidney disease patients who do not use gadolinium is urgently required.

Though not yet ready for clinical translation, oxygen supply imaging with hyperpolarized MR contrast agents are techniques with exceptional promise in precisely these areas of clinical need. Like H_2_^15^O PET and unlike gadolinium-based MR contrast agents, hyperpolarized agents directly generate MR signal, so hyperpolarized MR signal intensity is proportional to tissue concentration and therefore tissue perfusion. When imaged with MR, the lack of background signal means that acquired images show only structures containing the infused contrast agent. Imaging the distribution of a non-metabolized hyperpolarized agent is a theoretically simple, direct and repeatable method for quantitative angiography and/or perfusion mapping to demonstrate any defects in the oxygen supply [12].

### Imaging oxygen supply in cardiovascular disease

Several proof-of-concept studies have provided evidence that hyperpolarized contrast media may be useful to achieve MRA. Hyperpolarized ^13^C-urea was infused via a catheter after selective intubation of the left coronary artery in healthy pigs, and the coronary arteries were imaged with a balanced steady-state free-precession (b-SSFP) acquisition sequence [30]. As an initial proof-of-concept study, the results were promising. However, the images were acquired with a projection imaging technique (slice thickness, 15 cm), and the in-plane spatial resolution (2 × 2 mm) was an order of magnitude away from that achievable with CT. The temporal resolution of the images was 422 ms, still some way from that achievable with CT (approximately 80 ms). Since then, the temporal resolution of hyperpolarized ^13^C MRA data acquisition methods has increased to 291 ms [31], with similar spatial resolution also achieved using a b-SSFP sequence and projection imaging techniques.

Contrast-enhanced techniques that monitor the first-pass tracer kinetics of gadolinium chelates through the tissue of interest are the most commonly employed to measure tissue perfusion. In the heart, contrast-enhanced MRI has been validated against gold-standard invasive measurements including fractional flow reserve (FFR) [32], and authors have repeatedly found that cardiac perfusion MRI is a sensitive and specific method for detecting stress-inducible ischaemia and is useful to guide revascularization decisions. However, quantification of MR perfusion images remains time consuming, and many centres continue to rely on visual image analysis. Quantitative analyses may be helpful to identify stress-inducible ischaemia in patients with balanced hypoperfusion or three-vessel disease [12,32]. Moreover, there is an urgent clinical need to assess the functional significance of patients presenting with angina but no evidence of obstructive CAD and FFR above the threshold to indicate revascularization [33].

A number of investigators have demonstrated the feasibility of imaging ^13^C distribution to map perfusion. The proof-of-concept was performed by Golman and Petersson [30], who infused hyperpolarized ^13^C-urea intravenously into a pig, and acquired images with a b-SSFP pulse sequence and 3 × 3 mm in-plane spatial resolution with 10 mm slice thickness. Upon occlusion of the left anterior descending artery, no signal was measured in the corresponding coronary territory. Furthermore, the authors calculated quantitative myocardial perfusion maps on a pixel-by-pixel basis using the Kety–Schmidt method [34] after correcting for hyperpolarized tracer signal decay.

**Figure 2:**
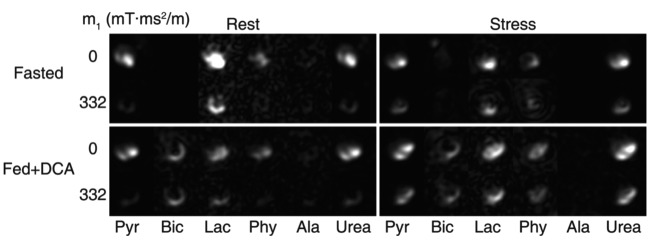
*In vivo* data showing hyperpolarized pyruvate, its downstream metabolites (bicarbonate, lactate, pyruvate hydrate and alanine) and urea in the rat heart. Each set of images corresponds to one combination of perfusion and metabolic state. Images are shown with either no flow encoding (top row) or flow sensitization (bottom row). The images are cropped to a 27  ×  27 mm^2^ field of view. Reproduced from [36] with permissions from the authors and publisher.

In the rodent heart, Lau et al. [35] demonstrated first-pass perfusion imaging with hyperpolarized ^13^C-urea and flow-sensitizing gradients that reduced ^13^C signal in the cardiac chambers, thus improving contrast with ^13^C-urea in the capillary bed. In a subsequent publication, the sequence was used upon co-polarization/administration with hyperpolarized [1-^13^C]pyruvate to simultaneously achieve metabolic and perfusion imaging in the absence and presence of hyperaemic stress (Figure 2, [36]). As concluded by the authors, the ability to rapidly measure metabolism/perfusion mismatch could have great clinical importance in guiding revascularization decisions in an acute setting (PET metabolism/perfusion mismatch studies have limited clinical utility because of logistics: they require a dual bolus of radioactive tracer, one each of ^18^F-FDG for metabolism and ^13^N-ammonia for perfusion and scan times approaching 1 h) [12]. Furthermore, regionally enhanced [1-^13^C]lactate production could emerge as a novel marker of the functional significance of a perfusion defect: if oxygen supply is reduced to a level low enough that it affects myocardial substrate metabolism by enhancing glycolysis, that would be the ultimate indication that reduced tissue perfusion has affected cardiac physiology. Measuring stress-inducible [1-^13^C]lactate production in the context of perfusion mapping is poised to offer a new strategy for guiding treatment decisions in atypical patients: angina in the absence of obstructive CAD and conditions in which balanced hypoperfusion and microvascular dysfunction are purported to play a role.

Finally and of great interest is to develop new agents ideally suited to probe perfusion. The freely diffusible agent perdeuterated ^13^C-labelled 2-methylpropan-2-ol was hyperpolarized using DNP [37] to a polarization level near 10%, with promising relaxation characteristics (*ex vivo* T1 of approximately 45 s and T2 of approximately 0.55 s at 9.4 T). This agent offers the essential benefit of a favourable and well-characterized toxicity profile.

### Renal perfusion imaging

Robust imaging of nutrient and oxygen supply to the kidney presents an urgent clinical need, both because of the organ’s unique physiology and the simple fact that many kidney patients requiring image-based assessments are the same patients who are at risk when given gadolinium-based contrast. Today, many patients with renal impairment do not get the optimal MR examination due to the risks associated with injection of gadolinium [38]. The essential renal tubular sodium transport is the primary determinant of renal oxygen consumption and even under normophysiological conditions the kidney oxygen tension is low [39,40]. Blood flow to the renal cortex normally supplies oxygen far in excess of its metabolic needs; however, blood flow to the medulla is low and there is considerable diffusion of oxygen from the arterial to the venous side resulting in the medulla often functioning in a hypoxic state. Increasingly, experimental evidence is implicating renal hypoxia as a unifying mechanism in acute kidney injury, chronic kidney disease and diabetic nephropathy [39,41,42].

In some cases of non-complicated anatomy of the renal artery, measurements of blood flow can be performed without exogenous contrast, using phase-contrast imaging (PC-MRI) [43,44]. Non-invasive, quantitative assessment of kidney perfusion can be performed without exogenous contrast using arterial spin labelling (ASL) [38]. However, as PC-MRI and ASL are subtraction techniques, a major challenge to renal applications (which is not faced in brain imaging) is movement arising from respiratory motion. Further developments to simplify post-processing of kidney ASL data will be required before widespread adoption of the technique in the clinic [38].

**Figure 3:**
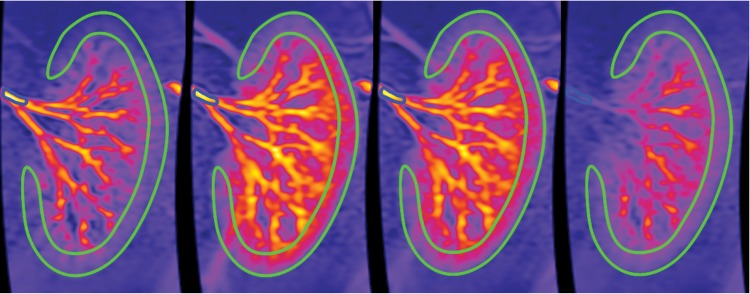
Image intensity in the arterial input region of interest (ROI) and cortex ROI for the same pig, upon infusion of hyperpolarized water. Images visualize time steps at 3, 5, 7 and 9 s. Reproduced from [46] with permissions from the authors and publisher.

Ardenkjær-Larsen et al. [45] demonstrated that high-resolution, high contrast angiography can be performed using hyperpolarized water initially in rats, and recently in pigs [46]. Protons dissolved in deuterium oxide (^2^H_2_O) were hyperpolarized using DNP to a level of >5%, with a T_1_ of 24 s, and the hyperpolarized water was injected into the renal artery of a pig. A low flip angle gradient echo sequence was used to perform high-contrast dynamic imaging (Figure 3), and renal cortical perfusion was quantified to be approximately 504 ml/100 ml per min, a value in close agreement with that measured using contrast-enhanced methods.

The ability to hyperpolarize water protons in a polarizer designed for clinical use, with sufficient polarization to allow ultrafast angiographic and perfusion assessment, shows great promise for future clinical translation [46]. Imaging of hyperpolarized protons allows for the use of coils and pulse sequences already available in the clinical setting. The magnetization achievable with hyperpolarized water is superior to other nuclei, because of its large gyromagnetic ratio and the high proton concentration. Furthermore, as a tracer for perfusion, hyperpolarized water could have additional interesting properties. Water diffuses freely in the vascular bed and between cells, potentially providing information not obtainable with larger gadolinium-based agents.

The agents hyperpolarized ^13^C-urea and a molecule known as HP001 (bis-1,1-(hydroxymethyl)-[1-^13^C]cyclopropane-d8, an efficiently polarized exogenous compound with a long T1 of 95 s *ex vivo*, 32 s *in vivo* at 3 T) have been used to demonstrate perfusion in liver and kidney tissue in mice with high spatial resolution (0.038 cm^3^ voxel size) [47,48]. An advanced b-SSFP sequence was developed with a variable flip angle to maximize hyperpolarized signal available from each tracer. The use of hyperpolarized ^13^C-urea as a perfusion agent to interrogate the kidney is particularly intriguing, due to the presence of dedicated urea transporters in the kidney [49,50] that are coupled to the urine concentration mechanism. As the primary determinant of oxygen consumption is the sodium transport, or in other words the regulation of the osmolality gradient by handling of sodium, chloride and urea [51], detailed modelling of ^13^C-urea kinetics in the blood vessels, renal cortex and renal medulla is of great interest for studying the intra-renal oxygen needs. Hyperpolarized ^13^C-urea has been demonstrated to simultaneously assess both kidney perfusion and function during both acute intervention and during the progression of kidney diseases in both rodent and large animal models resembling human physiology, and warrants further exploration in humans [52–55].

## Imaging oxygen consumption and utilization

### Physiological role of O_2_

The physiological mechanisms in place to deliver the right amount of oxygen to a tissue target a simple goal: enabling oxygen to serve as the electron acceptor in the electron transport chain, the last step of the oxidative phosphorylation process that directly generates ATP. In principle, the amount of oxygen consumed by a tissue correlates with the amount of energy generated by that tissue, and oxygen consumption should reflect tissue workload. In practice, however, depending on the tissue in question, different oxygen utilization efficiencies among metabolic substrates, the occurrence of anaerobic (glycolytic) metabolism that can produce a small amount of ATP, and changes to the efficiency of ATP utilization in various pathophysiological states mean that oxygen consumption can become uncoupled from functional capacity of a tissue.

As such, the direct and non-invasive assessment of substrate metabolism represents a vital parameter in assessing the health of any tissue, yet remains a challenge for all imaging modalities. In the brain, overall rates of oxygen consumption can nearly be quantified without radiation by implementing T2-relaxation-under-spin-tagging (TRUST-MRI) methods [56], but very few attempts to measure the metabolic rate of O_2_ consumption in other organs have even been attempted in animal studies, let alone in the clinic. Furthermore, methods such as ^31^P and ^17^O MRS capable of assessing oxidative metabolism are inherently limited by low signal and have not penetrated into routine clinical practice [57–62]. Finally, although PET and MR measurements provide overall rates of oxygen consumption/energy production, there is no technique with capability to follow individual pathway contributions to energy metabolism within clinically relevant scan times. Interrogating the balance between carbohydrate, fatty acid, amino acid and ketone, metabolism in oxidative ATP production is essential to understand mechanisms underlying metabolic disease, and potentially to identify candidates for metabolic therapy [59]. And this is where the power of hyperpolarized ^13^C MR lies – the ability to interrogate mechanisms of abnormal substrate oxidation, rapidly and non-invasively.

### Assessing oxidative substrate metabolism using hyperpolarized ^13^C MR

The majority of work in the field of hyperpolarized MR, in applications in non-oxidative (tumour) and oxidative tissue, has utilized hyperpolarized [1-^13^C]pyruvate to interrogate the balance among the major ATP-producing pathways: anaerobic glycolysis, glucose oxidation and fatty acid oxidation [27,63–66]. By shifting the ^13^C label to the second carbon of pyruvate, real-time TCA cycle metabolism has also been followed in the heart and brain with hyperpolarized MR [67,68]. Furthermore, the metabolism of hyperpolarized ^13^C-glucose has been followed in tumours through glycolysis and into the pentose phosphate pathway [69,70]. The metabolic fate of this tracer has yet to be explored in oxidative tissue but could yield insight into the kinetics of glucose uptake and glycolytic–oxidative coupling, particularly in diabetes and ischaemia.

Progress has been made in the direct detection of fatty acid oxidation using hyperpolarized MR. Physiologically, the majority of fatty acids available to fuel energy metabolism are in the form of long chain fatty acids, such as palmitate, bound to albumin in the blood. Large molecules such as palmitate inherently have fast T1 relaxation, which makes the use of them as hyperpolarized tracers difficult; what complicates their use even further is that T1 relaxation upon binding to albumin is sub-second [71]. Thus, efforts to polarize short and medium chain fatty acids, such as acetate, butyrate and octanoate, are currently the only methods that have been successfully visualized *in vivo* [72–74]. While outside the scope of the present study, it is worth mentioning the potential relevance of imaging brown fat and the ‘browning’ process using hyperpolarized short-chain fatty acids, particularly [1-^13^C]acetate [75].

Finally, a key aspect to measuring substrate oxidation is the ability to assess reduction–oxidation (redox) potential in tissue; specifically, monitoring aberrant mitochondrial production of reactive oxygen species (ROS) that has been implicated in numerous pathophysiological processes. Developments towards the goal of measuring tissue redox potential and ROS have been made using hyperpolarized MR. For example, when hyperpolarized dehydroxyascorbic acid is infused *in vivo*, the resultant dehydroxyascorbate/ascorbate (DHA/VitC) ratio appears to depend on redox-coupled (via the NADPH oxidases) glutathione activity [76–78]. The rate of [1-^13^C]lactate labelling following infusion of hyperpolarized [1-^13^C]pyruvate itself has indexed availability of cytosolic reducing equivalents (i.e. NADH) [79–81]; interestingly, the [1-^13^C]lactate-to-[1-^13^C]pyruvate conversion is sensitive to modulation of intracellular ROS themselves [82]. Finally, efforts to integrate the ^13^C-hyperpolarized label into molecular imaging-type probe design platforms have led to promising *in vitro* probes with sensitivity to specific ROS, although these tracers have yet to be refined for *in vivo* use [83,84].

### Targeting cardiovascular metabolism using hyperpolarized ^13^C MR

To meet its task of continually circulating blood throughout the body, the heart consumes more ATP than any other organ [59]. The healthy heart derives 60–90% of its energy from the oxidation of fatty acids, with the remainder primarily from pyruvate oxidation, derived from glucose (via glycolysis) and lactate. However, when plasma substrate composition is altered, the relative contributions of lipids, carbohydrates and ketone bodies to cardiac acetyl-CoA and subsequently oxidative ATP production vary substantially.

*In vivo* control of the PDC enzyme complex is a fundamental determinant of the relative contributions of glucose and fatty acid oxidation to ATP production in the heart [85]. Several PDC-mediated mechanisms exist to promote fatty acid oxidation over glucose oxidation, including phosphorylation-inhibition and end-product inhibition. The primary application of cardiac hyperpolarized ^13^C MR has been to monitor flux of [1-^13^C]pyruvate through the PDC to form ^13^CO_2_ and ^13^C-bicarbonate, the first non-invasive measurement which has been confirmed to demonstrate the *in vivo* operation of the glucose–fatty acid cycle [63,64]. In pathophysiological states including fasting, high-fat and ketogenic diets, diabetes, dilated cardiomyopathy (DCM), hypoxia and hypothyroidism, flux through the PDC have been demonstrated by hyperpolarized ^13^C to be reduced [63,64,86–92]. Complementary *in vitro* assays demonstrated these reductions were due to increased phosphorylation. In cases of dobutamine stress or elevated workload due to pathological hypertrophy [93,94], PDC has been elevated, showing an increased contribution to ATP production from carbohydrates. Pharmacological intervention studies with PDC activator dichloroacetate (DCA) have demonstrated that in certain cases, normalizing metabolic phenotype can prevent or revert the development of cardiac remodelling [87,91]. Analysis of the production rate of ^13^C-bicarbonate from ^13^CO_2_ in intact hearts has revealed that metabolically generated CO_2_ must be actively vented from the mitochondria by the enzyme carbonic anhydrase (CA), to preserve the proton-gradient driving oxidative ATP production [95].

Monitoring cytosolic conversion of [1-^13^C]pyruvate into [1-^13^C]lactate has been shown to reflect an increased reliance on glycolytic ATP production in anaerobic states (hypoxia and/or ischaemia) or diseases in which glycolysis and glucose oxidation are uncoupled [93,96]. Imaging [1-^13^C]lactate has shown promise in delineating damaged, yet viable tissue in infarction models [97] and may be useful in determining the functional significance of a perfusion defect. Pre-clinical data and data from cancer patients suggest that subtle shifts towards [1-^13^C]lactate production may be a biomarker of chronic, low-grade hypoxia and/or inflammation that characterize immunometabolic disorders such as HFpEF. Modelling the uptake and metabolism of hyperpolarized glucose [69] may also reveal impairments to metabolic flexibility in hibernating myocardium, heart failure or diabetic cardiomyopathy. A combined approach using hyperpolarized ^13^C-pyruvate and ^13^C-glucose could help to clarify the conflicting results from several recent clinical trials, in which administration of glucose-lowering drugs to heart disease patients seems to improve metabolic parameters yet have disparate effects on cardiac function [98–100].

**Figure 4:**
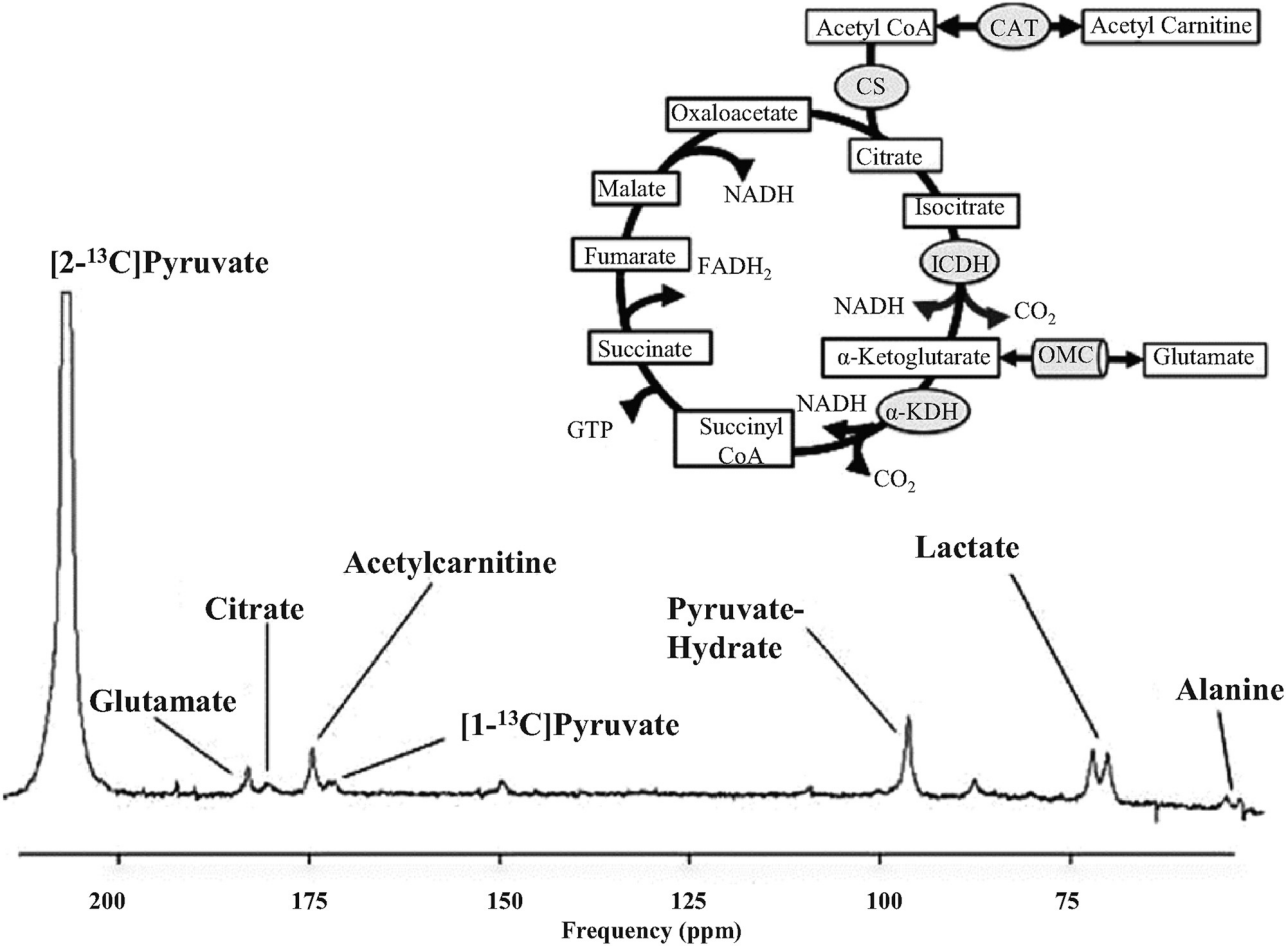
Representative spectrum acquired from a rat heart *in vivo* after infusion of hyperpolarized [2-^13^C]pyruvate. This metabolic tracer has enabled *in vivo* assessment of first-pass TCA cycle dynamics. CAT indicates carnitine acetyltransferase; CS, citrate synthase; ICDH, isocitrate dehydrogenase; OMC, oxoglutarate–malate carrier and αKDH, α-ketoglutarate dehydrogenase. Reproduced from [12] with permissions from the authors and publisher.

**Figure 5:**
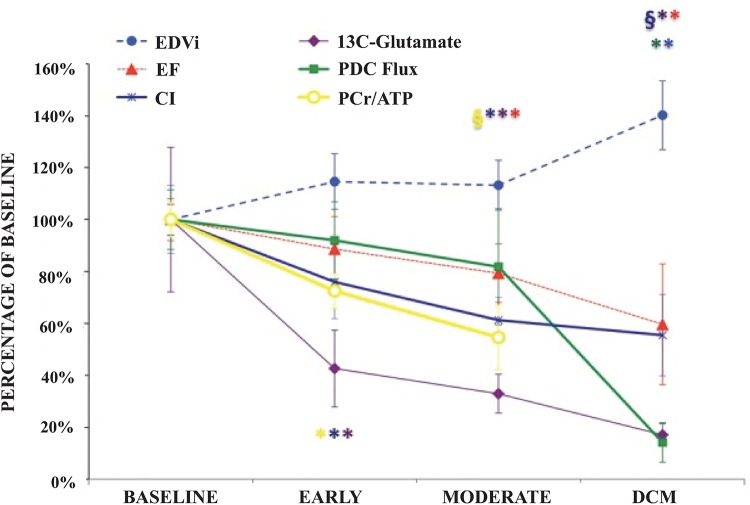
An overview of LV metabolic, energetic, structural and functional remodelling, measured non-invasively using MRI and MRS, throughout the development of tachycardia-induced DCM. Altered first-pass TCA cycle dynamics, as evidenced by reduced [5-^13^C]glutamate production, was among the earliest changes observed with the development of disease; *P<0.05 compared with baseline; ^§^*P*<0.05 compared with the early time point. CI, cardiac index; EDVi, end-diastolic volume index; PCr, phosphocreatine. Reproduced from [92] with permissions from the authors and publisher.

When hyperpolarized [2-^13^C]pyruvate is used as a metabolic tracer, the ^13^C label is retained within acetyl-CoA rather than being released as ^13^CO_2_, enabling downstream metabolic steps to be visualized (Figures 4 and 5). The enzymatic conversions of hyperpolarized [2-^13^C]pyruvate to [2-^13^C]lactate, [1-^13^C]acetyl-carnitine, [1-^13^C]citrate and [5-^13^C]glutamate were observed with sub-second temporal resolution in the perfused [67] and *in vivo* [94] rat heart. Hyperpolarized [2-^13^C]pyruvate has revealed reduced flux through the TCA cycle acutely during reperfusion [67] and chronically during cardiac remodelling as a result of myocardial infarction [101]. The tracer revealed altered oxidative metabolism in a porcine model of DCM before any functional or structural changes to the myocardium could be detected [92]. In our mind, it is particularly interesting to consider the disparate mechanism by which TCA cycle kinetics could affect ^13^C accumulation in the glutamate and citrate pools. For example, pathologically elevated workload – as may be the case in the porcine DCM model and indeed in the human hypertrophic heart – would increase TCA cycle flux, preventing ^13^C export into the glutamate pool [102], whereas impaired first-pass TCA flux could cause the same effect on ^13^C-glutamate accumulation via an opposing mechanism. Further work modelling the kinetics of [2-^13^C]pyruvate metabolism through the first span of the TCA cycle [103] is warranted to understand the mechanisms of observed changes in terms of pathophysiology, and to determine if altered first-pass TCA cycle kinetics could be useful as a biomarker of cardiac dysfunction.

To interrogate fatty acid metabolism directly would be beneficial in cardiac research. Of particular interest is a study in which [1-^13^C]pyruvate and [1-^13^C]butyrate were co-polarized and co-injected into fed and fasted rats, to study myocardial substrate selection using hyperpolarized MR [88]. As expected, in the fasted state pyruvate oxidation was reduced alongside increased butyrate oxidation and hyperpolarized labelling of ketone bodies by pseudoketogenesis. A co-polarization approach shows great potential to interrogate metabolic flexibility and obtain a more comprehensive ‘metabolic fingerprint’ in disease, reflecting competitive metabolism through multiple pathways. Also intriguing is the possibility that the acetyl-CoA synthetase-mediated hyperpolarized [1-^13^C]acetate to [1-^13^C]acetyl-CoA conversion may form the basis of an assay for intracellular CoA levels [12,72].

Imaging oxidative stress using hyperpolarized MR in the myocardium remains an unexplored area, though one of great clinical significance [79]. Depletion of reduced glutathione and increased NADPH oxidase levels (in particular Nox2 and Nox4) are known to cause detrimental oxidative stress during acute MI and in heart failure [104,105], suggesting a role for ^13^C-DHA imaging in the ischaemic heart. Comparing the time courses of the ^13^C-DHA/VitC and ^13^C-lactate/^13^C-pyruvate ratios during the onset of ischaemia and during reperfusion injury, alongside biomarkers of cell death such as plasma troponin levels or hyperpolarized ^13^C-fumarate mediated measurements of necrosis [106], may form the foundation for a new method to assess myocardial viability and guide treatment decisions. Also of interest would be application of redox tracers to study diabetic cardiomyopathy, and HFpEF where oxidative stress has been proposed as a unifying mechanism of disease [3,107]. In both cases, the ability to evaluate redox status could be valuable in evaluating the efficacy of potential therapies. Furthermore, the high rate of mitochondrial respiration characteristic of cardiomyocytes implies that different types of ROS are fundamental determinants in both physiological signalling and pathological disease [108]. Collaborations between chemists working to develop molecular imaging-type approaches with ROS-sensitivity and a ^13^C label, and cardiologists with the means and capacity to test these tracers *in vivo* in experimental models of ischaemia/reperfusion, HFpEF and the cluster of pathologies contributing to metabolic syndrome, could yield enormous benefit in understanding and treating cardiovascular diseases.

### Imaging kidney metabolism using hyperpolarized ^13^C MR

Hyperpolarized MR studies of the kidney have focused primarily on interrogating metabolic changes indicative of hypoxia or oxidative stress, the two initiating insults that are widely proposed as the potential unifying mechanisms for kidney disease [39,40,109,110]. In the diabetic kidney, increased [1-^13^C]lactate production has been measured, consistent with the presence of intra-renal pseudo hypoxia [111–113]. The importance of oxygen availability in the diabetic kidney was demonstrated by our research group, when we observed an additional increase in [1-^13^C]lactate and [1-^13^C]alanine production (likely indicative of increased [1-^13^C]pyruvate uptake) in diabetic kidneys inspiring reduced oxygen levels typical of high altitude [112]. Moreover, in the presence of suboptimal insulin administration, kidneys demonstrated increased aerobic and anaerobic metabolism ([1-^13^C]lactate and ^13^C-bicarbonate production), suggesting that poor glycaemic control places an additional burden to maintain renal energy metabolism and oxygenation without affecting the metabolic phenotype [111]. In an ischaemia-reperfusion mediated model of AKI, [1-^13^C]lactate production was elevated 24 h after reperfusion to a degree correlating with reductions in kidney function, indicating that the hyperpolarized metric could offer an imaging biomarker of tubular injury in the renal cortex [114].

Hyperpolarized tracers, in particular [1-^13^C]pyruvate and [2-^13^C]dehydroxyacetone, may also clarify the kidney’s role in gluconeogenesis in various pathophysiological states. The kidney is still often overlooked as an important gluconeogenic organ, with primary focus on the liver. However, awareness of renal mechanisms of glucose homoeostasis is likely to increase in the near future because novel glucose-lowering drugs are being developed that inhibit the renal sodium–glucose co-transporter 2 (SGLT2) [115]. The hyperpolarized tracer [2-^13^C]dehydroxyacetone enters gluconeogenesis at the level of the trioses [118], and has shown potential in the perfused liver [118] and in the liver and kidneys of intact rats to monitor opposing gluconeogenic and glycogenolytic fluxes [119]. Furthermore, following infusion of hyperpolarized [1-^13^C]pyruvate in the fasted and diabetic kidneys and liver, increased production of the gluconeogenic precursor lactate has been observed. This occurred in concert with increased expression of phosphoenolpyruvate carboxykinase (PEPCK), a key regulator of gluconeogenesis [116]. In a study of diabetes-induced cardiorenal syndrome (CRS), increased [1-^13^C]lactate production following infusion of hyperpolarized [1-^13^C]pyruvate was detected in both heart and kidney. Expression of the genes encoding regulatory enzymes PEPCK (*Pck1*) and glucose-6-phosphatase (*G6pc)* were both increased in kidney whereas only *Pck1* expression was increased in heart, a non-gluconeogenic organ. Both of these sets of results suggested that hyperpolarized ^13^C MR may be valuable in assessing systemic processes (exemplified by gluconeogenesis) in which various organs have distinct roles [117].

Hyperpolarized ^13^C-DHA was used in a murine model of diabetes to assess renal redox capacity [78]. At an early stage of disease, the diabetic mice demonstrated a decreased capacity to reduce DHA to VitC, correlating with lower tissue reduced glutathione concentration and higher Nox4 expression, and consistent with an increased burden of oxidative stress. Treatment with an angiotensin-converting enzyme (ACE) inhibitor restored the reduced glutathione levels and Nox4 expression, thus normalizing ^13^C-DHA/VitC ratio. The authors concluded that hyperpolarized ^13^C-DHA enabled non-invasive assessment of redox capacity in diabetic renal injury and in response to treatment [78]. Oxidative stress is also the purported mechanism driving the CRS, and as such, progressive monitoring of the DHA/VitC ratio in models of primary and secondary CRS could provide important information regarding the interplay of the two organs [117].

## Future directions and challenges

The development of any new imaging technique, and its incorporation into clinical practice, relies on the fact that the technique can provide information that cannot be reliably obtained by any other method and that improves patient outcomes. In many areas of imaging oxygen metabolism, hyperpolarized MR methods have shown potential to solve urgent clinical needs. Now that it has been demonstrated that hyperpolarized MR methods can be implemented to image patients, the next steps will be to confirm that hyperpolarized MR can solve these clinical needs by providing information about disease that is unique. To achieve this, from the perspective of imaging oxygen metabolism:

1. Conceptual advancements to enable imaging absolute tissue oxygenation should be pursued;
2. Technological improvements to requisite hardware and software will be required to increase patient throughput and improve data quality;
3. Kinetic modelling and data quantification must be improved;
4. New contrast media, particularly those tailored for angiographic and perfusion applications, must be developed for clinical use; and
5. Comprehensive pre-clinical and clinical hyperpolarized studies must be designed that extend hyperpolarized MR methods beyond the proof-of-concept stage and pinpoint unique benefits to patient outcome.

### Imaging tissue oxygenation

The oxygenation status of a tissue represents the balance achieved between oxygen supply via the blood perfusion, and metabolic oxygen demand. Thus, no discussion of oxygen metabolism is complete without analysis of the methods available to assay for tissue oxygen levels.

The success and widespread research application of blood oxygen level dependent (BOLD) MRI are such that BOLD imaging in the brain has become virtually synonymous with the concept of ‘imaging tissue oxygenation.’ In the brain, kinetic models accounting for blood flow, blood volume, haemoglobin oxygen saturation, biological parameters including vessel geometry and pH and magnetic field homogeneity have been developed to advance BOLD-MRI to a quantitative technique (qBOLD) for cerebral oxygenation. The clinical impact of renal and cardiac BOLD, however, has been reduced by long acquisition times, low signal to noise, an associated lack of robustness that is also the result of cardiac and respiratory motion and the need for standardized analysis [4].

Recently, our group demonstrated that investigating changes to the relaxation mechanism of hyperpolarized tracer may offer a robust, rapidly implemented solution such that the key parameter of oxygenation may also be assessed in the heart and kidney. A single shot radial fast spin echo (FSE) sequence with golden ratio encoding was implemented, and the sequence was used to map the T2 relaxation of hyperpolarized ^13^C-urea in the kidneys of control and diabetic rats. Region of interest analysis was performed to separate the renal segments into the cortex, medulla and papilla. In all animals, a significant intra-renal gradient across segments was identified reflecting the intra-renal distribution pattern of urea, with the lowest T2 relaxation found in the cortex and the highest in the pelvis. Interestingly in healthy kidneys, T2 relaxation of hyperpolarized ^13^C-urea correlated with peripheral capillary oxygen saturation measurements. T2 relaxation was significantly shorter in diabetic kidneys than in healthy kidneys, and moreover, that the renal T2 was uncoupled from peripheral oxygen saturation in diabetics. Based on what we know about MR sensitivity to paramagnetic compared with diamagnetic blood and the results from this proof-of-concept study, it is tempting to speculate that T2 relaxation of hyperpolarized ^13^C-urea is directly dependent on the local oxygenation status of renal tissue. Further work is warranted to pinpoint the determining factors towards T2 relaxation of hyperpolarized media, which likely include tissue oxygenation as well as structural changes, and to move towards quantitative tissue characterization based on this rapid and high-signal measurement. Hyperpolarization of [^13^C,^15^N_2_]urea may have a key role in the development of this technique, due to the multiple order-of-magnitude increases in T2 conferred by the ^15^N label. The lengthened T2 translated into enhanced signal in an SSFP acquisition, with flip angles approaching the fully refocused regime [120].

There is no question that BOLD-MRI measurements of oxygenation in kidney and heart, limited as they are, have made a significant impact on clinical research and raised fascinating technical and clinical questions. In the kidney, for example, BOLD-MRI has confirmed abnormal oxygen supply and/or oxygenation occurs in unilateral ureter obstruction [121,122], tissue hyperglycaemia, hypertension and CKD [4,123,124]. Correlations between cortical rates of deoxygenated haemoglobin, male gender, glycaemia and uric acid levels suggest that these factors interfere with the regulation of renal tissue oxygenation and warrant exploration with hyperpolarized methods [123]. In ischaemic heart disease patients, BOLD-MRI showed decoupling between angiographic defects, perfusion defects and oxygenation itself [125]. The authors suggested that there may be a physiological reserve or adaptive processes occurring in the face of reduced oxygen supply, such that perfusion may decrease within a safety margin without deoxygenation to the point of biochemical consequence. Furthermore, BOLD showed a higher ‘false-positive’ rate than did perfusion imaging (12% compared with 5%). This could have resulted from signal artifacts and low signal intensity on the BOLD images, or could represent the underlying physiology. The extension of hyperpolarized methods and BOLD-MRI techniques to reduce artifacts and improve volumetric coverage are warranted to continue exploration of these questions. Moreover, comparison of local hyperpolarized [1-^13^C]lactate production with measurements of tissue oxygenation could clarify the existence of an oxygenation reserve to buffer myocardial tissue against temporary oxygen supply/demand imbalances.

### Technological improvements

The technical advancements to the clinical hyperpolarizer (SpinLab) hardware that will be required for high-throughput patient studies are actively underway. Improvements in the ability to consistently produce sterile doses of hyperpolarized [1-^13^C]pyruvate, and to assess/confirm their sterility, are being performed on an ongoing basis and for the longer term. Projecting forward one year, it is reasonable to expect that >10 sites worldwide will be performing clinical hyperpolarized MR studies with [1-^13^C]pyruvate in cancer and oxygen metabolism applications combined, and the growth rate of the field is set to increase further as the technology required to produce sterile hyperpolarized agents becomes simpler, more robust and in turn more affordable. The major MR scanner vendors have adapted their products to facilitate the addition of hardware and software tailored to the ^13^C nucleus, and the researchers who have led the way in developing custom MRI and MRS pulse sequences for hyperpolarized applications have been extremely generous in assisting with their implementation across imaging platforms.

### Data quantification and the development of new tracers

Quantification of instantaneous metabolic fluxes will be essential for future development of both basic science and clinical applications of hyperpolarized MR. Basic science studies should strive both to measure metabolic fluxes in units that can be compared with conventional biochemical assays (i.e. μmol•min^−1^•g^−1^), as described in detail in [16]. Moreover, the metabolic flux parameters extracted from hyperpolarized MR images and spectra should be standardized such that studies can be effectively compared among all research sites [126]. In the clinic, a system for monitoring the effects of the input function of the hyperpolarized agent on metabolic images must be developed because tracer pharmacokinetics could differ dramatically between control subjects and patients [12,92]. Furthermore, modelling the physiological significance of ^13^C fluxes through dynamic metabolic pools, such as those associated with the CoA pool and TCA cycle in health and disease, will be essential to validate biomarkers (i.e. in heart failure) and use them to guide therapeutic decisions. Correlating ^13^C measurements with complementary PET metrics of substrate uptake and oxygen consumption, and MR studies of high-energy phosphate metabolism (^31^P MRS) and oxygen availability (BOLD), to understand how biochemical changes assayed with hyperpolarized MR affect overall oxygen consumption and energetics, will be useful to understand the functional significance of altered substrate metabolism and thus translating ^13^C into clinical application.

In the case of oxygen supply, comparisons between hyperpolarized parameters and quantifiable gold standard measurements, such as H_2_^15^O perfusion PET, FFR, ASL or first-pass gadolinium will be essential to validate that hyperpolarized tracer flow can be measured quantitatively and could therefore have a role in quantitative measurement of perfusion and towards using [1-^13^C]lactate production to assess functional significance of perfusion defects. To move towards quantification, non-metabolized perfusion tracers that are safe for intravenous infusion and retain hyperpolarized signal must be developed and their diffusion and relaxation kinetics must be characterized. Currently, the most promising potential candidates include hyperpolarized ^13^C-urea, perdeuterated ^13^C-labelled 2-methylpropan-2-ol and hyperpolarized water.

### Strategies for future study design

Many investigators working with hyperpolarized ^13^C MR may have heard variations on the following statements: (1) ‘That seems like a really expensive assay for PDC flux,’ and/or (2) ‘So hyperpolarized lactate production is up – again?’ There is no question that much of the proof-of-concept work in the field was oriented towards demonstrating changes, non-invasively, to single enzymes and that because of the finite number of energy producing pathways that exist in all tissues, there were only a few potential outcomes that could be detected. It is interesting to consider, for example, that many investigators (ourselves included!) have invested considerable resources in demonstrating changes to PDC flux in disease that are of lesser magnitude than those that occur in an overnight fast. But these ‘limitations’ to the technology are not really limitations at all – rather they reflect the early stage of many hyperpolarized ^13^C MR studies, and the vital role that balancing glycolysis, glucose oxidation and fatty acid oxidation to generate sufficient ATP plays in the regulation of nearly all tissues in the body. Essentially, hyperpolarized MR to assess substrate metabolism is a simple non-invasive enzyme assay, and the power of this technology to advance our ability to manage diseases of oxygen metabolism will depend on strategic pre-clinical and clinical study design.

In the basic science realm, mechanistic studies that consider the unique benefits of hyperpolarized MR (assaying tissue biochemistry *in situ*, spatial localization and high temporal resolution) alongside the technique’s finite capabilities and areas of unexplored physiology are advancing our understanding of human health and disease. For example, studies targeting the CA enzyme using hyperpolarized ^13^C-bicarbonate and/or hyperpolarized [1-^13^C]pyruvate [89,95,127,128], assessing acetylcarnitine buffering kinetics [72,94,129] and monitoring urea transport in kidney disease [49,50,52–55,130] have been particularly insightful in their conception and results. The results from these studies are poised for development into new diagnostic methods and have pinpointed new targets for therapy. Indeed, although strictly outside the scope of the present study, studies using hyperpolarized ^13^C-α-ketoglutarate to target aberrant accumulation of the oncometabolite 2-hydroxyglutarate in isocitrate dehydrogenase-1 mutant glioma [131] and using hyperpolarized [1,4-^13^C_2_]fumarate to detect necrosis *in vivo* [106] also fall into the category of strategic design and are illustrative to mention.

It is immediately obvious why application of hyperpolarized [1-^13^C]pyruvate MRI for prostate cancer diagnosis, based on Warburg-mediated pyruvate to lactate exchange, has generated such enthusiasm among the world’s top cancer and MRI research centres. Yet the ‘killer application’ [16] in measuring oxygen metabolism may not be so clear. This may be due to the metabolic flexibility of the myocardium, which enables the heart to adaptively generate ATP from any fuel source available in response to physiological stimuli. In other words, changes to cardiac substrate metabolism measured correctly by hyperpolarized MR often do not reflect disease. Strategic study design can compensate for this, however, by controlling metabolic state of the subject before scanning (as is performed in PET and renal BOLD imaging [4]) and by analysing hyperpolarized metabolic changes in context of other measurement methods. MR is lauded by many as a ‘one-stop shop’ for imaging structure and function [9,38] and moving forward, hyperpolarized MR will have the greatest benefit to human health if we use it as one tool in an imaging toolbox to examine the functional significance of oxygen metabolism. In their recent *Initial Experience* publication showing results from their first cohort of human subjects [14], Cunningham and colleagues indicated that the exemplary images they acquired from a hyperpolarized [1-^13^C]pyruvate scan added approximately 10 min to a clinical cardiac MRI protocol (Figure 1) [14]. This time scale makes it entirely feasible to incorporate hyperpolarized metabolic scanning with MR measures of cardiorenal perfusion, function and in future perhaps oxygenation, alongside more routine echocardiographic and blood-borne biomarker examinations. Typically, clinical cardiac MRI protocols already consider results from multiple scanning protocols [132], and this ‘one-stop shop’ approach is also becoming more prevalent in kidney research [38,44,52,112,121]. In this context, ‘increased local lactate production’ or ‘globally decreased PDC flux’ are metrics that could carry weight in pointing to a diagnosis.

Finally, the most compelling results to emerge from hyperpolarized MR may be on the horizon: for in oxidative tissues, many of the clinical questions for which the technology is best suited to address can only truly be evaluated in patients. Experimental models of myocardial infarction as well as acute kidney injury, for example, mimic ischaemia and reperfusion but cannot reproducibly take into account confounding factors that occur in patients: mechanical crushing and fragmentation of the culprit lesion upon percutaneous coronary intervention (PCI), no-reflow phenomenon, subtleties in arterial and structural anatomy and the occurrence of co-morbidities such as age, hypertension and metabolic syndrome. To our knowledge no animal models are widely accepted for chronic angina or angina in the absence of CAD, and HFpEF models that reproduce the diverse characteristics of the patient population are notoriously difficult to achieve. In HFpEF and other immunometabolic diseases that have become a worldwide epidemic, the unique ability of hyperpolarized MR to simultaneously measure metabolism among multiple organs and elucidate coupling of their functions could extract information currently unavailable [133]. Furthermore, assessing of substrate utilization during the anaerbobic to aerobic transition in human skeletal muscle [72], as well as the kinetics of acetylcarnitine buffering [94,129] and insulin resistance, will become illuminating when performed in people exercising within the MR scanner.

## Conclusion

Every tissue in the body critically depends on meeting its energetic demands with sufficient oxygen supply, and conversely, oxygen supply/demand imbalances underlie the diseases that inflict the greatest socio-economic burden globally. The maturation of hyperpolarized MR has provided the foundation for new non-invasive methods to measure tissue oxygen metabolism as part of a ‘one-stop shop’ evaluation of organ structure and function. Pre-clinical studies have indicated that by using hyperpolarized tracers with advanced MRI and MRS pulse sequences, it may be possible to measure blood supply, mechanisms of oxidative metabolism and tissue oxygenation rapidly and robustly, with high signal generated by safe contrast. We expect the impact to be greatest in assessing the heart and the kidney, organs in which oxygen requirements are particularly stringent and which present imaging challenges that have not been met by current technology. The recent translation of hyperpolarized MR methods into the clinic will hasten the technological refinements and patient research required to cement its role as a clinical tool for assessment of oxygen metabolism.

## Abbreviations

AKI: acute kidney injury
ASL: arterial spin labeling
ATP: adenosine triphosphate
BOLD: blood oxygen level dependent
b-SSFP: balanced-steady state free precession
CA: carbonic anhydrase
CAD: coronary artery disease
CKD: chronic kidney disease
CRS: cardiorenal syndrome
CT: computed tomography
DCM: dilated cardiomyopathy
DHA: dehydroxyascorbic acid
DNP: dynamic nuclear polarization
FFR: fractional flow reserve
HFpEF: heart failure with preserved ejection fraction
LDH: lactate dehydrogenase
MI: myocardial infarction
MR: magnetic resonance
MRA: MR angiography
MRS: magnetic resonance spectroscopy
PDC: pyruvate dehydrogenase enzyme complex
PEPCK: phosphoenolpyruvate carboxykinase
PET: positron emission tomography
ROS: reactive oxygen species
TCA: tricarboxylic acid
TRUST: T2-relaxation-under-spin-tagging
